# Personalising adherence-enhancing interventions using a smart inhaler in patients with COPD: an exploratory cost-effectiveness analysis

**DOI:** 10.1038/s41533-018-0092-8

**Published:** 2018-06-27

**Authors:** Job F. M. van Boven, Breda Cushen, Imran Sulaiman, Garrett Greene, Elaine MacHale, Matshediso C. Mokoka, Frank Doyle, Richard B. Reilly, Kathleen Bennett, Richard W. Costello

**Affiliations:** 1 Department of General Practice & Elderly Care, University Medical Center Groningen, Groningen Research Institute for Asthma and COPD (GRIAC), University of Groningen, Groningen, The Netherlands; 2 Department of Pharmacy, Unit of Pharmacoepidemiology & Pharmacoeconomics, University of Groningen, Groningen, The Netherlands; 3 Royal College of Surgeons in Ireland, Clinical Research Centre, Smurfit Building Beaumont Hospital, Dublin, Ireland; 4 Department of Psychology, Division of Population Health Sciences, Royal College of Surgeons in Ireland, Dublin, Ireland; 5 Trinity Centre for Bioengineering, Trinity College, The University of Dublin, Dublin, Ireland; 6 Division of Population Health Sciences, Royal College of Surgeons in Ireland, Dublin, Ireland; 7 Department of Respiratory Medicine, Royal College of Surgeons in Ireland, Dublin, Ireland

## Abstract

Four inhaler adherence clusters have been identified using the INCA audio device in COPD patients: (1) regular use/good technique, (2) regular use/frequent technique errors, (3) irregular use/good technique, and (4) irregular use/frequent technique errors. Their relationship with healthcare utilization and mortality was established, but the cost-effectiveness of adherence-enhancing interventions is unknown. In this exploratory study, we aimed to estimate the potential cost-effectiveness of reaching optimal adherence in the three suboptimal adherence clusters, i.e., a theoretical shift of clusters 2, 3, and 4 to cluster 1. Cost-effectiveness was estimated over a 5-year time horizon using the Irish healthcare payer perspective. We used a previously developed COPD health-economic model that was updated with INCA trial data and Irish national economic and epidemiological data. For each cluster, interventions would result in additional quality-adjusted life years gained at reasonable investment. Cost-effectiveness was most favorable in cluster 3, with possible cost savings of €845/annum/person.

## Introduction

In chronic obstructive pulmonary disease (COPD), real-world adherence to maintenance therapy can be as low as 20%.^1^ In contrast, trials report adherence rates of over 80% in most participants.^2^ Previous studies showed that patients with high adherence have significantly better clinical and economic outcomes.^3^ Consequently, interventions focusing on adherence enhancement have shown to be effective and cost-effective.^4,5^ Yet, in these studies, only average adherence was assessed and no distinction was made between the different aspects of non-adherence. Optimal implementation of inhaled therapy involves both regular use as well as good inhaler technique.^6^ In our previous work, we identified four distinct clusters of inhaler adherence that were associated with differential clinical outcomes.^7^ Economic evaluations of adherence-enhancing interventions in each of those clusters have not been performed, but could help prioritizing specific target populations for tailored interventions. The aim of this follow-up study is to estimate the potential cost-effectiveness of reaching optimal adherence in each suboptimal adherence cluster.

## Results

**Fig. 1:**
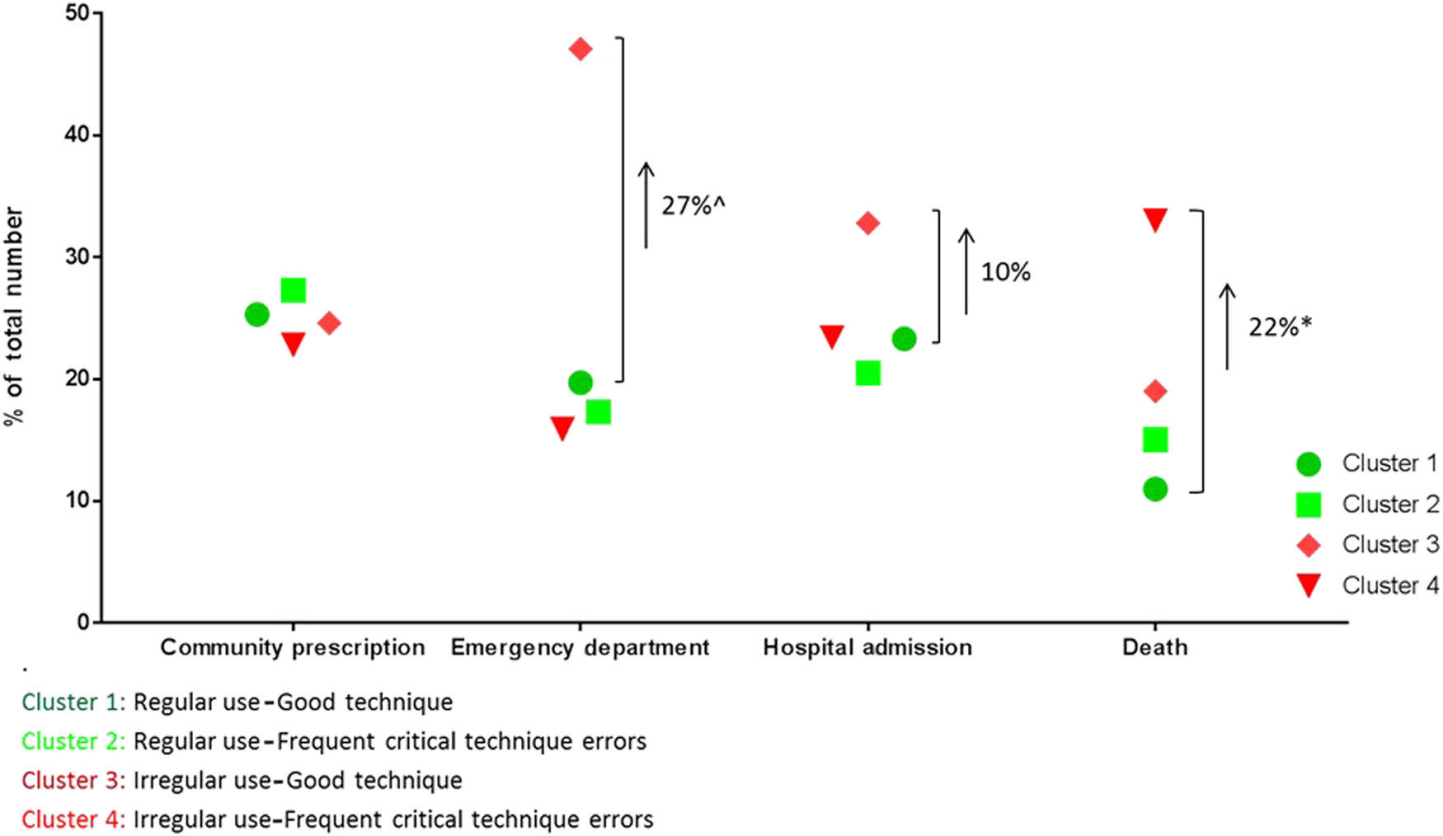
Proportional contribution of each adherence cluster to all-cause clinical outcomes over the 12-month follow-up period (adjusted for the number of participants per cluster). Reported differences are the absolute differences in the proportion of events attributable to cluster 3 vs. cluster 1 for emergency department and hospital admission and cluster 4 vs. cluster 1 for death. ^ denotes *p* = 0.05, *p < 0.05

The study’s baseline population characteristics (*n* = 226) have been described elsewhere.^1,7^ In short, cluster 1 had the lowest mortality during follow-up. Cluster 2 had the highest rate of antibiotic and/or oral steroids community prescriptions. The highest overall healthcare use was attributable to patients from cluster 3. Cluster 4 had the highest mortality (Fig. 1).

### Cost-effectiveness

Cost-effectiveness analysis results are shown in Table 1. Adherence-enhancing interventions seem generally cost-effective, given all ICERs are below the Irish cost-effectiveness threshold of €45,000/QALY.^8^ In other words, this means that all adherence-enhancing interventions would cost less than €45,000 to gain one life-year in perfect health. For each cluster, the theoretical intervention would result in additional life years and QALYs gained. Notably, for cluster 3, an intervention would be cost-saving (i.e., less costs and more QALYs).

**Table 1:** Cost-effectiveness of enhancing adherence based on a 5-year time horizon

Cluster	Total costs	Intervention costs^a^	Medication costs	Healthcare costs	Life years	QALYs
*Cluster 2: Regular use, frequent technique errors*						
Intervention	€11,386	€184	€2901	€8300	3.75	3.00
Cluster 2	€10,150	€0	€2723	€7428	3.52	2.81
Difference	€1235	€184	€179	€872	0.23	0.19
ICER	€6520/QALY gained					
*Cluster 3: Irregular use, good technique*						
Intervention	€11,812	€184	€2936	€8692	3.80	3.03
Cluster 3	€12,657	€0	€2395	€10,262	3.40	2.70
Difference	−€845	€184	€541	−€1570	0.39	0.33
ICER	Cost-saving (i.e., less costs, more QALYs)					
*Cluster 4: Irregular use, frequent technique errors*						
Intervention	€13,075	€184	€3000	€9891	3.88	3.10
Cluster 4	€10,180	€0	€1918	€8261	2.98	2.36
Difference	€2896	€184	€1082	€1630	0.90	0.74
ICER	€3935/QALY gained					

*ICER* incremental cost-effectiveness ratio, *QALY* quality-adjusted life year

^a^Note that the mean per-patient intervention costs are slightly lower given some patients die within the first year and so do not cost the full €200

## Discussion

We performed exploratory cost-effectiveness modeling to assess the potential economic benefits of improved adherence in three clusters of patients with suboptimal adherence. In all clusters, interventions seem cost-effective. Moreover, in patients with irregular use but good inhaler technique, a possible cost-saving of €845/annum/person could be yielded despite the higher cost of medication arising from better adherence.

Remote monitoring of adherence on a longitudinal basis provides an accurate evaluation of regularity of use as well as inhaler technique. The use of a technology, such as the one described in this report, identified four patterns of adherence and may give guidance as to how we might approach this challenge in a personalized manner. Hence, in the future, by providing personalized interventions to the highest risk groups we may both improve adherence and have a positive impact on both clinical and economic outcomes in those most in need, such as those in cluster 3. The need for personalized adherence-enhancing interventions has recently been highlighted.^9^ They could include education and training on inhaler technique for those with frequent technique errors, patient reminders for those with irregular use due to forgetfulness, and shared decision-making and motivational interviewing for those with irregular use due to a conscious, intentional decision.^9^

This study is a first attempt to estimate the cost-effectiveness of interventions in different adherence clusters, but was limited by the use of post hoc and short-term effectiveness data for the different clusters. Regarding generalizability, absolute costs may differ per country or setting, but we expect relative cluster results to be comparable. Our exploratory cost-effectiveness model estimates should be confirmed when long-term clinical intervention trial data become available, including extensive sensitivity and scenario analyses as well as analyses in a non-admitted primary care COPD population.

## Conclusion

Personalized adherence interventions targeting patient-specific regularity of inhaler use and inhaler technique could result in clinical and economic benefits for COPD patients.

## Methods

### Study design

This was an exploratory cost-effectiveness analysis. Ethical approval for the clinical study was obtained from the Beaumont Hospital Ethics Committee.

### Patient population

Patient selection and data collection has been described previously.^1^ Briefly, hospitalized patients with COPD, prescribed salmeterol/fluticasone propionate (Seretide®, GlaxoSmithKline, Ireland), were included and written informed consent was obtained from all participants.

### Inhaler adherence

Adherence was assessed using the INCA^TM^ device that can track both timing and quality of inhaler use.^1^ The INCA acoustic recordings allowed for identification of the following four adherence clusters:^7^ (1) regular use/good technique, (2) regular use/frequent technique errors, (3) irregular use/good technique, and (4) irregular use/frequent technique errors. Cluster-specific healthcare utilization and mortality was studied previously^7^ and is summarized in Fig. 1.

### Cost-effectiveness analysis

The cost-effectiveness of enhanced adherence, i.e., the theoretical shift of patients from poor adherence (i.e., cluster 2, 3, or 4) to good adherence (i.e., cluster 1), was estimated. Therefore, a previously developed, described, and validated health-economic model^5^ was used and populated with Irish and INCA-specific cost (for medication, intervention, and healthcare utilization) and effect (mortality and exacerbations) data (Appendix 1). The model has received a high-quality score in the latest COPD model review,^10^ and estimates the incremental cost-effectiveness ratio (ICER) in terms of costs (2013, €) per quality-adjusted life-year (QALY) gained from the Irish healthcare payer’s perspective assuming fixed per-patient intervention costs of €200 (authors’ estimation). Three ICERs were calculated using cluster 1 as intervention and clusters 2, 3, and 4, respectively, as usual care scenarios. In line with recommendations,^11^ a policy-relevant time horizon of 5 years was used, taking into account a 5% discount rate for both costs and effects as per Irish Health Information and Quality Authority guidelines (www.hiqa.ie).

### Data availability statement

Detailed data and characteristics of the study population are available in ref. ^1^ and other data that support the findings of this study are available from the corresponding author on reasonable request.

## Electronic supplementary material

Appendix 1: Input parameters for cost-effectiveness model
