# Supplementary material for: Personalising adherence-enhancing interventions using a smart inhaler in patients with COPD: an exploratory cost-effectiveness analysis
Source: NPJ Prim Care Respir Med. 2018 Jun 27;28:24. doi: 10.1038/s41533-018-0092-8 (PMC6021429; doi:10.1038/s41533-018-0092-8)
Supplement: Supplementary file 1 — Appendix 1: Input parameters for cost-effectiveness model [file 41533_2018_92_MOESM1_ESM.docx]

**Appendix 1: Input parameters for cost-effectiveness model**

| **Parameter** | **Value** | **Source** |
| --- | --- | --- |
| General |  |  |
| Baseline age (years) | 71 | [7] |
| Baseline FEV1% predicted | 51 |  |
| Baseline % male | 49 |  |
| Baseline % current smokers | 24 |  |
| Baseline exacerbation rate (in year 1, per 3 months) |  |  |
| *Cluster 2* |  |  |
| Mild COPD: Community treated | 1.625 | [7] |
| Mild COPD: ED treated | 0.125 |  |
| Mild COPD: Hospital treated | 0.000 |  |
| Moderate COPD: Community treated | 0.885 |  |
| Moderate COPD: ED treated | 0.033 |  |
| Moderate COPD: Hospital treated | 0.333 |  |
| Severe COPD: Community treated | 1.443 |  |
| Severe COPD: ED treated | 0.125 |  |
| Severe COPD: Hospital treated | 0.453 |  |
| Very severe COPD: Community treated | 1.150 |  |
| Very Severe COPD: ED treated | 0.150 |  |
| Very Severe COPD: Hospital treated | 0.000 |  |
| *Cluster 3* |  |  |
| Mild COPD: Community treated | 0.938 | [7] |
| Mild COPD: ED treated | 0.063 |  |
| Mild COPD: Hospital treated | 0.250 |  |
| Moderate COPD: Community treated | 1.110 |  |
| Moderate COPD: ED treated | 0.328 |  |
| Moderate COPD: Hospital treated | 0.720 |  |
| Severe COPD: Community treated | 1.133 |  |
| Severe COPD: ED treated | 0.400 |  |
| Severe COPD: Hospital treated | 0.300 |  |
| Very severe COPD: Community treated | 0.925 |  |
| Very Severe COPD: ED treated | 0.063 |  |
| Very Severe COPD: Hospital treated | 0.542 |  |
| *Cluster 4* |  |  |
| Mild COPD: Community treated | 0.165 | [7] |
| Mild COPD: ED treated | 0.000 |  |
| Mild COPD: Hospital treated | 0.083 |  |
| Moderate COPD: Community treated | 0.635 |  |
| Moderate COPD: ED treated | 0.115 |  |
| Moderate COPD: Hospital treated | 0.405 |  |
| Severe COPD: Community treated | 1.715 |  |
| Severe COPD: ED treated | 0.073 |  |
| Severe COPD: Hospital treated | 0.678 |  |
| Very severe COPD: Community treated | 1.600 |  |
| Very Severe COPD: ED treated | 0.050 |  |
| Very Severe COPD: Hospital treated | 0.200 |  |
| Intervention effects |  |  |
| *Cluster1 vs cluster 2* |  |  |
| RR community treated exacerbation | 0.92 | [7] |
| RR ED treated exacerbation | 1.13 |  |
| RR hospital treated exacerbation | 1.13 |  |
| Baseline adherence (%) – proportion of days covered | 78 |  |
| Adherence improvement by intervention (%) | 0 |  |
| *Cluster 1 vs cluster 3* |  |  |
| RR community treated exacerbation | 1.03 | [7] |
| RR ED treated exacerbation | 0.41 |  |
| RR hospital treated exacerbation | 0.71 |  |
| Baseline adherence (%)– proportion of days covered | 71 |  |
| Adherence improvement by intervention (%) | 7 |  |
| *Cluster 1 vs cluster 4* |  |  |
| RR community treated exacerbation | 1.11 | [7] |
| RR ED treated exacerbation | 1.22 |  |
| RR hospital treated exacerbation | 0.98 |  |
| Baseline adherence (%)– proportion of days covered | 65 |  |
| Adherence improvement by intervention (%) | 13 |  |
| All-cause mortality |  |  |
| Cluster 1 (proportion died in year 1) | 0.111 | [7] |
| Cluster 2 (proportion died in year 1) | 0.154 |  |
| Cluster 3 (proportion died in year 1) | 0.191 |  |
| Cluster 4 (proportion died in year 1) | 0.330 |  |
| Follow-up base risk (year 2-5) | Age/sex specific all-cause mortality | IPHI^a^ |
| RR intervention cluster 1 vs cluster 2 | 0.72 | [7] |
| RR intervention cluster 1 vs cluster 3 | 0.58 |  |
| RR intervention cluster 1 vs cluster 4 | 0.33 |  |
| Exacerbation rate (>1 year) |  |  |
| Mild COPD – Community treated | 0.61 | [5] |
| Mild COPD – ED treated | 0.06 |  |
| Mild COPD – Hospital treated | 0.11 |  |
| Moderate COPD – Community treated | 0.89 |  |
| Moderate COPD – ED treated | 0.08 |  |
| Moderate COPD – Hospital treated | 0.16 |  |
| Severe COPD – Community treated | 1.22 |  |
| Severe COPD – ED treated | 0.11 |  |
| Severe COPD – Hospital treated | 0.22 |  |
| Very severe COPD – Community treated | 1.55 |  |
| Very severe COPD – ED treated | 0.14 |  |
| Very severe COPD – Hospital treated | 0.28 |  |
| Costs (€, 2013) |  |  |
| Intervention per-patient per year | 200 | Authors’ estimation |
| Medication (100% adherence) per year | 991 | Irish national formulary |
| Exacerbation community treated | 130 (20) | Ready Reckoner^b^ |
| Exacerbation ED treated | 238 (40) | Ready Reckoner^b^ |
| Exacerbation hospital treated | 3873 (581) | Ready Reckoner^b^ |
| Discount rate costs | 5% | www.hiqa.ie |
| Utilities |  |  |
| Mild COPD state | 0.897 | [5] |
| Moderate COPD state | 0.755 |  |
| Severe COPD state | 0.748 |  |
| Very Severe COPD state | 0.549 |  |
| Exacerbation community treated | -0.0166 | [5] |
| Exacerbation ED treated | -0.0300 |  |
| Exacerbation hospital treated | -0.0482 |  |
| Discount rate effects | 5% | www.hiqa.ie |

COPD: chronic obstructive pulmonary disease; ED: emergency department; RR: relative risk

^a^Institute of Public Health Ireland http://data.thehealthwell.info/NTI/indicators/tables.php?resID=2073.

^b^Ready reckoner of acute hospital inpatient and daycase activity & costs (summarized by DRG) relating to 2011 costs and activity 2013.
